# Book Reviews

**Published:** 1914-07

**Authors:** 


### BOOK REVIEWS

PROGRESSIVE MEDICINE.—A Digest of the Advances, discoveries and improvements in the Medical Sciences. Edited by Hobart H. Ware, M. D. Assisted by L. F. Appleman, M. D. Vol. 1, March, 1914. Published by Leo & Febiger, Philadelphia.

This volume contains the following articles: Surgery of the Head and Neck, by C. H. Frazier, M. D.; Surgery of the Thorax, excluding Diseases of the Breast, by G. P. Muller, M. D.; Infectious Diseases, including acute Rheumatism, Croupous Pneumonia and Influenza, by John Rurhah, M. D.; Diseases of Children, by F. M. Crandall, M. D.; Rhinology and Laryngology, by Geo. B. Wood, M. D., and Otology, by A. R. Duel, M. D.

This is a splendid summary and digest of the work of the past year. One may read nothing but Progressive Medicine and yet keep well informed in the advances we are making.

---

THE CLINICS OF JOHN B. MURPHY, M. D., at Mercy Hospital, Chicago, February, 1914. Published bi-monthly by W. B. Saunders Co., Philadelphia.

This volume contains much concise and valuable information of the work done at this well-known clinic. Much of the space is devoted to bone and joint surgery, in which Murphy excels. A useful chapter is devoted to tuberculosis of the testicle. A talk is given by Crile on nitrous oxide anesthesia.

---

THE PATHOGENESIS OF SALVARSAN FATALITIES. By Professor Wilhelm Wechselmann, Berlin, Germany. Translated by Clarence Martin. M. D., St. Louis. Published by The Fleming-Smith Co., St. Louis, Mo.

Whether or not one subscribes to Wechselmann's views that the damage done to the kidneys by mercurial treatment increases the danger of salvarsan, he must admit that such a monograph as this must be of the greatest value to those who wish to treat syphilis with the least possible risk. He urges that an exact technic be followed; that the dose be accurately adapted to the individual case; that .exact chemical and microscopical examinations of the urine be made; that if the combined treatment be used, the salvarsan should be given first and the mercury later and finally that every general reaction following an injection of salvarsan should be carefully investigated to determine, if possible, its cause.

